# Trafficking of Vietnamese women and girls for marriage in China

**DOI:** 10.1186/s41256-017-0049-4

**Published:** 2017-10-09

**Authors:** Heidi Stöckl, Ligia Kiss, Jobst Koehler, Dung Thuy Dong, Cathy Zimmerman

**Affiliations:** 1 Gender Violence and Health Centre, Department of Global Health and Development, Faculty of Public Health and Policy, London School of Hygiene and Tropical Medicine, 15-17 Tavistock Place, London, WC1H 9SE UK; 2 IOM Vietnam, Ho Chi Minh, Vietnam

**Keywords:** Human trafficking, Vietnam, China, Forced marriage, Mental health and violence against women

## Abstract

**Background:**

Bride-trafficking has been a growing phenomenon in Southeast Asia, particularly in China, where one-child policies have resulted in demographic imbalances favoring males. Yet, empirical evidence about women and girls sold into marriage in China remains sparse.

**Methods:**

This study describes the experiences of 51 Vietnamese women and girls as young as 14 in post-trafficking services who were sold into marriage in China. A consecutive sample of individuals from five services in Vietnam were invited to be interviewed within the first 2 weeks of admission. It is part of a wider dataset for research on the health of men, women, and children in post-trafficking services in Cambodia, Thailand, and Vietnam, the largest study to date on human trafficking and health. We calculated proportions for all variables and conducted bivariate analyses using Fisher Exact Tests for their associations with mental health disorders symptoms.

**Results:**

Before leaving home, 31% (*n* = 15) participants were married. Participants reported high levels of sexual violence (*n* = 43; 86%) while trafficked and59% (*n* = 30) spent time in detention before returning to Vietnam. Once in post-trafficking care 52.9% (*n* = 27) reported probable depression, anxiety or post-traumatic stress disorders (PTSD), two women (4%) attempted suicide in the past month and 38 (75%) remained afraid of their trafficker. Ten (22%) became pregnant while trafficked, and seven (16%) were pregnant at the interview.

**Conclusions:**

The cross-border bride trade between Vietnam and China raises complex policy issues, including questions about detention and immigration rights and strategies for supported return processes, including maternal and child health services. As the repercussions of China’s absent female population will persist, Vietnam and China must grapple with the political and social conditions to implement prevention strategies, and resources for women who fall prey to this cross-border bride trade.

## Background

Human trafficking is a crime that involves extreme forms of exploitation, which often leaves survivors with enduring physical and psychological scars [1, 2]. The most commonly accepted definition of human trafficking is found in the UN Convention Against Transnational Organized Crime, which emphasizes that trafficking involves the coercion and/or deception of individuals for the purposes of exploitation, including for “sexual exploitation” and “slavery or practices similar to slavery.” [3]. While the term ‘slave-like practices’ in the protocol includes trafficking for marriage, there remains little empirical evidence or conceptual clarity about this abusive phenomenon [4], which is often only associated with the “mail order bride” business from low and middle income countries to North America and Europe [5].

The sale of women for marriage has a long history in China. After the Communist Party gained power in 1949, they instituted policies to redefine social, gender and family relationships, including abolishing rampant female trafficking and prostitution. As a result, there appeared to be no further officially recognized cases of trafficking in women [6, 7] until the introduction of the free market economy and the rapid rise in migration in the late 1970’s [6, 8]. The main underlying factors thought to influence the trafficking of women for marriage into China include patriarchal structures and China’s one-child policies. Patriarchal structures, such as the widespread expectation for men to marry and produce a male offspring for the continuation of the family line has met with a dramatic increase in bride prices since the pre-1978 era. As a result, Chinese men who are pressured by their family to marry, but lack sufficient financial or other assets, are often not considered socially desirable partners for women [9, 10].

The one child policy, paired with the cultural preference for sons, has resulted in a skewed sex ratio in rural China and its impoverished regions [11]. The number of marriageable women in rural Chinese villages has further declined because a large number of women have migrated to more developed coastal areas of eastern China for work or better marriage prospects [11]. The numerous men who are unable to find wives and the social problem this creates for their families receives considerable sympathy from villagers and cadres, who accept practices that involve the purchasing of a bride against the bride’s will and therefore would be unlikely to report them to police [11]. Additionally, in rural areas, when a marriage is arranged by parents, a bride-to-be’s consent may not be considered necessary and many will not see the difference between paying a ‘bride-price’ versus buying a wife. [8, 12].

In Vietnam, poverty, unemployment, patriarchal structures and a surplus of Vietnamese women in rural areas in the past due to years of war are tied to a cultural expectation for single women to marry, which seem to fuel the trade in women for marriage to China [11, 13].

The uneven socioeconomic development between China and Vietnam make cross-border activities and migration attractive, particularly because crossing the border only requires a boarder pass versus a passport [13]. Furthermore, in rural Vietnam, women are expected to marry at a relatively early age, yet because it can be difficult for Vietnamese women to find attractive husbands, marriage abroad is viewed as a viable option [13]. There is a history of marriage between Chinese men and Vietnamese or Burmese women, which sometimes includes bride-kidnapping, a practice often accepted by communities, leaving kidnapped brides to fend for themselves. [9, 11].

Despite its high profile in news reporting and international policies, empirical evidence – both quantitative and qualitative - on trafficking of women into forced marriage is scarce. One qualitative study investigates the trafficking of Bangladeshi girls as wives to North India, focusing on interviews with women trafficked ten to 20 years ago. This study illustrated the numerous ways children were sold knowingly or unknowingly into marriage, highlights their hard lives and their difficulties to reintegrate upon return—if they were able to leave [14]. Two other empirical studies, an ethnographic study and a quantitative survey of migrants, each with an unidentified number of women experiencing forced marriage explored the trafficking into forced marriage of North Korean women fleeing to China. None of the women in these studies planned to marry Chinese men but were rather recruited immediately after they crossed the border because of their likely vulnerability [10, 15]. A handful of studies also explore the “Mail order bride” business of women from Eastern European, Russian, and South East Asian to North America and Europe, however, most of this work relies on desk-based assessments websites, newspaper reports or legal cases rather than empirical work [5, 16]. To our knowledge, only two quantitative studies have examined the trafficking of women as wives from Vietnam to China, to date. The first study included 13 women who were trafficked for marriage [17], and the second study used respondent-driven sampling of women locally known as having been trafficked and included 213 women. However, the latter research initially included both trafficked women and any woman who had migrated to China, married and might have been trafficked. Therefore it is difficult to differentiate between trafficked women and those who migrated for marriage or to discern experiences of those who were trafficked [13].

Despite the high visibility of trafficking for marriage in newspapers and international reports, empirical evidence is rare. While reports have hypothesized about push and pull factors for bride-trafficking within and to China, there remains scant evidence on what women experience during these situations and what hopes and concerns they have after they return in terms of their health, well-being and families. This study is one of the first to provide the socio-economic characteristics of women trafficked into forced marriage and in post-trafficking services and to describe their experiences before, during and after their trafficking experience.

## Methods

This study is a sub-study of 51 Vietnamese women and girls as young as 14 in post-trafficking services in Vietnam who were sold into marriage in China. This sub-study uses data from a larger observational cross-sectional study investigating the health of men, women and children in post-trafficking services in Cambodia, Thailand, and Vietnam [1]. Within the larger study, trafficking for marriage was only observed among respondents from three post-trafficking services in Vietnam (40 women in the shelter Lao Cai, seven in the shelter Lang son and four in the shelter Peace House). Participants were a consecutive sample of clients of post-trafficking services. Trained interviewers conducted face-to-face interviews with all participants within their first 2 weeks of admission to the services after offering their informed consent. Individuals were excluded from the study if trained caseworkers deemed them too unwell to participate. The research teams followed a strict ethics protocol based on the WHO Ethical Recommendations for Interviewing Trafficked Women, ensuring voluntary and confidential participation, assurance that declining participation would not affect their access to services, minimization of distress and referral offers for health or other reported problems [18]. The International Organization for Migration Vietnam office coordinated the data collection and entry, with oversight by the London School of Hygiene and Tropical Medicine between October 2011 and May 2013. The study was approved by the ethics committee of the London School of Hygiene and Tropical Medicine and the Hanoi School of Public Health in Vietnam.

In the standardized survey, women were asked about their socioeconomic background, pre-trafficking exposures, experiences of violence, physical and mental health, and future plans and concerns. The questionnaire was translated into Vietnamese and refined through group discussions with International Organization for Migration counter-trafficking teams, further revised through pilot-testing, and reviewed after back-translation into English. It measured symptoms of anxiety and depression with the Hopkins Symptoms Checklist and post-traumatic stress disorder with the Harvard Trauma Questionnaire [19–21]. A cutoff of 1.75 was used for measuring anxiety [22] and 2.00 for post-traumatic stress disorder [23]. We excluded item 12 (sexual interest) from the depression scale because of sensitivity in cases of sexual abuse and because participants were often residing in shelter situations; therefore, we used 1.625 as the cutoff for symptoms indicative of depression, instead of the standard 1.75 cutoff [21] and assuming that each item made a similar contribution to the overall score. Physical and sexual violence was measured by a modified tool of the WHO international study of domestic violence [24] which has been supplemented by items victims of trafficking commonly report to local service providers. Participants were asked about health problems experienced in the past 4 weeks and variables were coded as positive for people who reported severe levels (“extremely” and “quite a lot”). Given the lack of empirical research, especially quantitative knowledge and tools, participants were also able to give open ended responses in addition to set response categories to questions concerning recruitment, reasons for leaving, return, reintegration, concerns and hope for the future. Qualitative quotes are used to illuminate the context of existing categories or to highlight the existence of different reasons or situations than those captured by the survey tool.

### Statistical analysis

The analysis is focused on women who were trafficked for marriage from Vietnam to China. Two women in the dataset who were trafficked within Vietnam and to Indonesia were not included into the following analysis. The analysis calculated proportions for all variables. Associations between symptoms of anxiety, depression and PTSD with specific aspects of their trafficking experience are assessed using Fisher exact tests. The analysis was conducted with Stata (Version 13). The study summarized the answers and used direct quotes given in the open-ended questions on reasons for leaving, hopes upon return and concerns.

## Results

Of the 51 women in this sample, 15 (29.5%) were minors at the time of the interview and 18 (36.7%) stated that they were already married before they were trafficked to become wives in China. Nineteen women (63%) had children at the time of the survey. Table 1 provides further details on participant’s socio-demographic characteristics. Sexual and/or physical violence was experienced by eight (16%) women prior to trafficking, with five women (10%) reporting to have been forced to have sex and five (10%) reporting physical violence.

**Table 1:** Socio-demographic characteristics of Vietnamese women trafficked for marriage to China

	No.	Percent
Age at the time of the interview		
< 15	2	3.9%
15–17	13	25.5%
18–24	21	41.2%
25–34	12	23.5%
> =35	3	5.9%
Educational level		
Primary (1–5 grade)	17	33.3%
Secondary (6–8 grade)	17	33.3%
Higher (10–11 grade)	3	5.9%
No formal schooling	10	19.6%
Missing	4	7.8%
Marital Status before leaving home		
Single, never married	24	49.0%
Married, but not living with spouse	3	6.1%
Married and living with spouse	15	30.6%
Separated or divorced	4	8.2%
Widowed	1	2.0%
Missing	2	4.1%
Children	19	63.3%
Employment prior to the trafficking experience		
Agriculture/farming/plantation	40	78.4%
Animal farming	1	2.0%
Car care	1	2.0%
Construction	1	2.0%
Factory work	1	2.0%
Manicure/nail care/hair wash	1	2.0%
Student	5	9.8%
Not working	1	2.0%

### Reasons for leaving, recruiter and trafficking knowledge before

Based on responses to the open-ended questions, it became clear that only one woman knew that she would marry a Chinese man before she left home. The other 50 women each believed they were agreeing to leave home for different reasons and the majority had not planned to migrate or even travel to China. Some women said they were recruited by being invited for a drink or a cup of water and then woke up in China, others were offered a job, travel or outing or simply a lift to a destination. Deception was a common recruitment tactic, as indicated by the following quotes:

“My lover deceived me to go to meet his parents”.

“A stranger induced me to go to China to buy clothes”.

“I was deceived to find a job in Lao Cai province”.

Women reported that they were deceived by: friends (*n* = 13); relatives, including for example, their aunt (*n* = 3), boyfriend (*n* = 2), and husband (*n* = 1); someone they knew (*n* = 2); and strangers (*n* = 12).

“My Vietnamese husband deceived me and trafficked me to China”.

The woman who reported that she was asked to marry in China stated later in the survey that she was not given accurate information about her situation there. The majority of the women (*n* = 44, 86%) never had identity or travel documents, and of the seven who did, five (71%) reported that the documents were confiscated during the trafficking situation.

Women who wanted to leave home opted to migrate because: they knew others who left and earned money (*n* = 9, 17.0%); they did not earn enough money in their job (*n* = 6, 11.3%); or could not find a job nearby (*n* = 3, 5.7%); or they needed money to support children (*n* = 2, 3.8%). Two women said they were abducted (3.8%). Other reasons included: to earn money to pay for medical care for ill family member; experiencing violence at home; no livelihood or house to live in; and having a boring life and seeking adventure.

Although most reported having been deceived by someone, more than half of the women blamed themselves for getting into the trafficking situation (*n* = 27, 53%). A few women also blamed others, including: their parents (2%); other family members (*n* = 5, 10%); a friend (*n* = 5, 10%); a boyfriend (*n* = 4, 8%); an acquaintance from the village (*n* = 5, 10%); an acquaintance not from home (*n* = 2, 4%); a broker (*n* = 7, 14%); and someone they did not know before (*n* = 33, 65%). Others were also blamed, such as: a husband; an agency; and a kidnapper. Half of the women (*n* = 25, 49%) reported they had heard about human trafficking before leaving home.

### Experiences and living conditions during the trafficking process

The majority of women were trafficked for more than a year (*n* = 33, 66%), with four women having been trafficked for two or more years. Seventeen women (34%) were trafficked for less than 100 days, with seven trafficked for less than 1 month. Women trafficked as brides were in the trafficking situation longer than individuals who were trafficked into other sectors (e.g., sex work, domestic work, fishing, factory work, etc) [1].

While all women in this study were trafficked to be wives, 11 women were also exploited in other sectors while in the trafficking process, including prostitution (*n* = 4, 8%), domestic work (*n* = 2, 4%), agriculture (*n* = 2, 4%), and cleaning and factory work (*n* = 1 in each, 2%). Only two of those 11 women (18%) were paid for this work. Only four women (8%) spoke Mandarin fluently, but it is unclear if they learnt the language during their trafficking experience or whether they were fluent beforehand.

Most women reported extremely restricted freedom, with 73% (*n* = 37) stating they were never free to do what they wanted or to go where they wanted and 13.7% (*n* = 7) said that they were ‘seldom’ free. For ten women (20%) restrictions were extreme, as they were physically locked in a room.

Nearly all women (*n* = 46, 90%) reported physical and/or sexual violence while in the trafficking situation, with 86% (*n* = 43) stating they were forced to have sex, including the two 14 year-old girls. A majority (*n* = 31, 61%) reported they were physically abused, as described in Table 2. Husbands were the main perpetrators of physical violence (*n* = 42, 91%) and sexual abuse (*n* = 40, 93%), followed by traffickers, with 41% (*n* = 19) perpetrating physical violence and 7 % (*n* = 3) sexual abuse. Two women (4.3%) reported physical violence by their employer. Additionally, women reported physical violence perpetrated by a client, a brothel security staff, a father-in-law and a motorbike taxi driver (*n* = 1 each, 2%). Perpetrators of sexual violence included: traffickers; clients (*n* = 3,7%); employers; brothel security staff; and a father-in-law (*n* = 1 each, 2.3%).

**Table 2:** Acts of physical violence and threats of violence experienced during the trafficking situation

	No.	Percent
Did anyone threaten to hurt you?	25	49%
Did anyone threaten to hurt your family or someone you care about	6	12%
Slapped you, shoved you or threw something at you that could hurt you	19	37%
Pushed or shoved you	17	33%
Hit you with a first or with something else that could hurt you	16	31%
Kicked, dragged or beat you up	16	31%
Tied or chained you	5	10%
Choked you on purpose	5	10%
Threatened to use a gun, knife or other weapon against you	9	18%
Used a knife to cut you	2	4%

### Escaping and end of the trafficking situation

The majority of women (*n* = 43, 84%) had attempted to escape the trafficking situation. Women were able to leave their circumstances because they either: ran away and escaped (*n* = 36 71%); got help from neighbors or people in the surroundings (*n* = 6, 12%); or were released by their husband or his family (*n* = 3, 6%). In this study, 42 (82%) women received help from the police, border guards or government officers. One woman also recounts that other trafficked wives worked together to flee:

“I contacted two people who were in the same situation and we planned to escape”.

It is unclear at what stage the police help the women, as it is notable that 30 women (59%) spent time in detention before returning home, with five (10%) spending up to 7 days, 11 (22%) between 8 and 32 days, nine (18%) between one and 3 months, and five (10%) more than 3 months in detention.

Eight women indicated that they did not try to escape, explaining they were: afraid of being killed (*n* = 5); feared revenge and violence (*n* = 4); feared arrest (*n* = 3); did not know the language (*n* = 3); and were afraid of getting lost (*n* = 3). Two women reported that they were harmed when they tried to leave previously, two did not have identity documents and three were prevented from leaving the compound because they were locked in a room or confined. Two women were also deterred because they had no money or prospect of livelihood upon return (*n* = 2), one feared harm to her families if she left (*n* = 1) and another feared she would be kidnapped to become a sex worker (*n* = 1). One woman did not want to leave her Chinese children behind.

### Health post-trafficking

Of the 51 women, 24 (47%) had symptoms of depression at the time they were interviewed, 15 (29%) reported symptoms of anxiety and seven (14%) symptoms of PTSD. Two women (4%) reported having tried to commit suicide in the previous month and one (2%) harmed herself physically. As presented in Table 3, symptoms of depression, anxiety and PTSD are significantly associated with experiences of physical violence during the trafficking experience. Women are also more likely to show symptoms of anxiety and PTSD if they were locked into a room while trafficked.

**Table 3:** Associations with mental health symptoms

	Symptomatic for depression					Symptomatic for anxiety disorder					Symptomatic for PTSD based on DSM 16 items score				
	No		Yes			No		Yes			No		Yes		
	N	%	N	%	*P*-value	N	%	N	%	*p*-value	N	%	N	%	*p*-value
Spent more than 100 days in trafficking situation	17	63.0%	16	69.6%	0.425	26	72.2%	7	50.0%	0.124	30	68.2%	3	50.0%	0.326
Held in detention	14	51.9%	16	66.7%	0.216	18	50.0%	12	80.0%	0.045	25	56.8%	5	71.4%	0.384
Locked in a room	4	14.8%	6	25.0%	0.287	1	2.8%	9	60.0%	<0.001	6	13.6%	4	57.1%	0.021
Free to do/go as wanted	18	66.7%	19	79.2%	0.248	25	69.4%	12	80.0%	0.343	33	75.0%	4	57.1%	0.287
Pregnant now or was pregnant during the trafficking situation	6	22.2%	5	20.8%	0.588	9	25.0%	2	13.3%	0.300	10	22.7%	1	14.3%	0.526
Physical violence during trafficking	11	40.7%	20	83.3%	0.002	17	47.2%	14	93.3%	0.002	24	54.5%	7	100%	0.023
Forced sex during trafficking	20	76.9%	23	95.8%	0.062	29	82.9%	14	93.3%	0.0311	36	83.7%	7	100%	0.323
Any violence during trafficking	22	81.5%	24	100%	0.034	31	86.1%	15	100%	0.160	39	88.6%	7	100%	0.462

In the 4 weeks prior to the interview, participants reported experiencing severe (“quite a lot” or “extremely”) physical pain or illness, including: dizzy spells (*n* = 9, 18%); headaches (*n* = 11, 22%); dental problems (*n* = 1, 2%); nausea or indigestion (*n* = 7, 14%); back pain (*n* = 12, 24%); skin problems (*n* = 4, 8%); extreme exhaustion (*n* = 4, 8%); memory problems (*n* = 2, 4%); and persistent coughing (*n* = 1, 2%).

At the time of the interview, three women (6%) aged 15, 16 and 19 said they never have had sex and did not experience sexual violence during their trafficking situation. Seven (16%) women stated that they were pregnant at the time of the interview and ten (22%) women reported a pregnancy during their trafficking situation. Seven women stated they had an intended termination of a pregnancy during the trafficking situation.

### Future plans and concerns

When asked about their future plans or worries, most women stated that they would like to live with their parents or other members of their family of origin after leaving the shelter (*n* = 39, 77%).

“I want return home to reunite with my family and then find a job to have money for my child’s studying”.

Nine women (18%) said they want to live with their former spouse, one explaining for example:

“I want to return home to live with my husband and children, have a job and bring up my children”.

One woman wanted to live at her work location and the others did not know.

The fear of their traffickers and their associates (*n* = 38, 75%) was one of women’s main concerns, stating, for instance

“Fearing the trafficker come back to retaliate against me”.

Fear was followed by feelings of guilt or shame (24%, *n* = 12); worries about earning money and having a job to pay debts (*n* = 11, 22%); their own mental health (*n* = 11, 22%); and their physical health (*n* = 8, 16%). A strong sense of shame and fear of traffickers has been previously described in a qualitative study of women returning from wife trafficking in China, because being trafficked to China is associated with sex work, loss of virginity and forced marriages in Vietnam [25]. Three women also mentioned that their main concerns were their children and husbands in China, about whom they are either thinking or to whom they want to return, with one woman planning to obtain the necessary legal documents to live with their husband and children in China noting:

“I want to return to live with my parents and ask for legal documents to come back to China to live with my husband and children in China”.

The remainder of the women clearly saw their future in Vietnam, reporting hopes such as: securing a job (*n* = 34); going home (*n* = 33); earning enough money (*n* = 18); bringing up their children and unborn babies (*n* = 17); studying and obtaining vocational training (*n* = 11); marrying and having a family (*n* = 9); living with their parents (*n* = 6); being able to support their families (*n* = 3); and buying a home (*n* = 1).

“I hope to have money, have family and job to bring up my coming baby (the victim is pregnant)”.

## Discussion

To the best of our knowledge, our findings represent the largest study sample to date of women trafficked for forced marriage from Vietnam into China and one of the only studies that specifically investigated survivors’ health. The characteristics of women in this study are similar in terms of age, educational and employment status to those of Duong’s et al.’s study of Vietnamese women who were considered to be trafficked in their community and consisted of women who migrated voluntarily or were trafficked into China for marriage [13]. In our study, half of the women (*n* = 24, 49%) were single and had never been married before they left home. In comparison, in a study of North Korean women trafficked for marriage to China, there was a higher percentage of women who were married and had children before they were trafficked [10]. The rate of physical and/or sexual violence (16%) reported by the women pre-departure is lower than the prevalence rate of 32.7% for physical and/or sexual violence found in a population-based study by Vung et al.’s [26] in rural Vietnam of 32.7%. This difference might be due to the relatively young age of women in our study.

Unlike previous studies, women in this study were mainly deceived and did not know that they would be married to a Chinese man. Reasons for this difference might be the different sampling strategies. In Duong et al., women were also recruited into the study who explicitly went to China for marriage but found the situation there to be very different to what they expected or had been promised, highlighting the difficulty of making a clear cut distinction between a woman who is considered trafficked and a woman who finds herself in an abusive marriage to which she initially agreed [13]. In the study by Blanchet et al. about Bangladeshi girls trafficked to North India for marriage, not all girls knew that they would be ‘sold’ into marriage, although the majority of them were aware that they would be married to a North Indian man. In addition, it was mainly their parents who had agreed to their marriage, as they were still under marriage age. Among women fleeing North Korea to China, none seem to have known that they would be trafficked and sold into marriage when they decided to leave North Korea [10, 27]. This is in stark contrast to women who participate in mail order bride businesses to Western countries, who agree to marry a foreign spouse initially, but might become trapped in abusive marriages [5]. The profile of traffickers described by women in this study is similar to that of other studies, as traffickers were frequently female and close or distant relatives or from the same social network as the trafficked women or complete strangers [13].

While they were trafficked, women in this study experienced extremely high levels of violence (90%), including sexual violence, mainly by their husbands and traffickers. Other research has also highlighted this, noting the extremely high prevalence of physical and sexual abuse that women experienced by their Chinese husbands and families. They attributed this to the fact that many Chinese men who resort to marrying trafficked women suffer from mental health problems, aggressive behavior, and substance abuse [10, 13]. North Korean women who were forced to marry Chinese men reported that husbands exerted additional control by threatening to report disobedient wives to immigration police, well-aware that the women feared repatriation and harsh—potentially fatal—consequences for themselves and their families in North Korea [27]. The studies of Korean women trafficked to be wives have also highlighted the systematic abuse by traffickers who rape and physically assault women to control them and make them compliant. Women are restricted in their response as they are anxioius to avoid detection by the Chinese police [10]. Nonetheless, it was interesting to find that a few trafficked wives in this study managed to escape the trafficking situation because they were released by their husbands. This is not entirely unusual, as other research has suggested that some Burmese women who were trafficked to be wives in China were permitted by their Chinese husbands and his family to return to Burma after the birth of their first child [9].

It was not uncommon for women in this study to be pregnant or leave behind a child or children in China, a fact that worried many of them. Duong et al.’s study explains in detail the legal complications associated with having a child that is not registered as Vietnamese [13], while other studies add that Chinese authorities do not repatriate Chinese born children of North Korean women when they are sent back [15] or highlight the potential stigma upon return when women had been trafficked into marriages with a partner of a different faith [14]. Trafficked women have to cope with these issues upon return, in addition to potentially strong symptoms of depression, post-traumatic stress disorder and anxiety, plus physical pains and illnesses, as identified in this study.

The findings of this study have to be considered in light of several limitations. Although this study represents the largest sample size of a study investigating trafficking for marriage, the sample size was still too small to investigate significant patterns of association beyond descriptive analyses. As human trafficking is a criminal activity, its scope is difficult to explore and representative samples nearly impossible to achieve. The study is based only on clients of post-trafficking services, inclusive only of women who managed to return to Vietnam and receive assistance by a shelter. Data were not collected directly from women in China, which poses an important limitation that should be addressed in future research. The study was also limited because instruments to measure mental health symptoms were not diagnostic and have not been validated with trafficked wives before, although all scales had a high reliability for all three outcomes. Also, the study relied on self-reported data from women trafficked for marriage. Answers could therefore be influenced by the wish to give socially desirable answers, as well as shame about having been deceived into these situations. As this study was part of a larger study on human trafficking, some aspects could not be explored and need to be investigated in future studies on wife trafficking, such as to which locations in China women were trafficked or if women left children behind. The responses women gave to open-ended questions suggest the limitations of current survey tools, which need to be further developed to gain a greater understanding of this subpopulation.

Nonetheless, our findings point to important issues that have rarely been considered in discussions on wife trafficking, such as women’s particular social and health care needs after being trafficked for forced marriage. Medical assessment, especially psychological support, should be included in all post-trafficking services. Services should also consider types of support women who opt to return home might want to foster safe and sustainable reintegration. Our study also indicates that special assistance (e.g., health, legal) might be needed for women who are pregnant or have had children with their Chinese husbands. Importantly, intervention research is urgently needed to identify feasible and effective psychological support approaches to help women recover from their complicated past and look forward to a more promising future. Their children will also need targeted support as some of them may be stateless, having been born in China without registration.

## Conclusion

There can be little doubt that bride-trafficking is, at its core, a profound expression of gender discrimination. It is a criminal abuse that is driven by deeply embedded social norms that accept, even promote, the concept that women can be treated as commodities. While our research clearly shows the need to support women who fall prey to this crime, these findings also strongly call out for sustained efforts to shift current gender norms towards the equal value and participation of women in society.
